# Optimization of Metronidazole Emulgel

**DOI:** 10.1155/2013/501082

**Published:** 2013-01-08

**Authors:** Monica Rao, Girish Sukre, Sheetal Aghav, Manmeet Kumar

**Affiliations:** Department of Pharmaceutics, AISSMS College of Pharmacy, Kennedy Road, Maharashtra, Pune 411 001, India

## Abstract

The purpose of the present study was to develop and optimize the emulgel system for MTZ (Metronidazole), a poorly water soluble drug. The pseudoternary phase diagrams were developed for various microemulsion formulations composed of Capmul 908 P, Acconon MC8-2, and propylene glycol. The emulgel was optimized using a three-factor, two-level factorial design, the independent variables selected were Capmul 908 P, and surfactant mixture (Acconon MC8-2 and gelling agent), and the dependent variables (responses) were a cumulative amount of drug permeated across the dialysis membrane in 24 h (*Y*_1_) and spreadability (*Y*_2_). Mathematical equations and response surface plots were used to relate the dependent and independent variables. The regression equations were generated for responses *Y*_1_ and *Y*_2_. The statistical validity of the polynomials was established, and optimized formulation factors were selected. Validation of the optimization study with 3 confirmatory runs indicated a high degree of prognostic ability of response surface methodology. Emulgel system of MTZ was developed and optimized using 2^3^ factorial design and could provide an effective treatment against topical infections.

## 1. Introduction

When gels and emulsions are used in a combined form the dosage forms are referred to as emulgels [1, 2]. As the name suggests they are the combination of emulsion/microemulsion and gel. In recent years, there has been great interest in the use of novel polymers with complex functions as emulsifiers and thickeners because the gelling capacity of these compounds allows the formulation of stable emulsions by decreasing surface and interfacial tension and at the same time increasing the viscosity of the aqueous phase. In fact, the presence of a gelling agent in the water phase converts a classical emulsion into an emulgel. Both oil-in-water and water-in-oil emulsions are used as vehicles to deliver various drugs to the skin [3]. Emulsions possess a certain degree of elegance and are easily washed off whenever desired. They also have a high ability to penetrate the skin. Emulgels for dermatological use have several favorable properties such as being thixotropic, greaseless, easily spreadable, easily removable, emollient, nonstaining, water soluble, having longer shelf life, biofriendly, transparent, having and pleasing appearance [4].

Several antifungal and antibacterial agents are available in the market in different topical preparations (e.g., creams, ointments, and powders for the purpose of local dermatological therapy). One of these antibacterial agents is Metronidazole (MTZ), which has antibacterial properties. MTZ is used for the treatment of *acne vulgaris*, skin lesions, wound drainage, and wound odor. MTZ possesses poor water solubility and hydrophobicity; hence such drugs pose problems in a topical drug delivery. Hence, for solubilization of MTZ, formulation of microemulsion-based gel appeared to be a viable approach. 

In the development of emulgel dosage form, an important issue is to design an optimized formulation with an appropriate drug diffusion rate in a short period of time and minimum number of trials. Many statistical experimental designs have been recognized as useful techniques to optimize the process variables. For this purpose, a computer-based optimization technique with a 2-level factorial design utilizing a polynomial equation has been widely used. This technique requires minimum experimentation and time, thus is far more effective and cost-effective than the conventional methods of formulating emulgel dosage forms. The aim of this investigation was to develop an emulgel system of MTZ using xanthan gum as a gelling agent and optimization of the formulation by applying the 2-level factorial design.

## 2. Materials and Methods

Metronidazole was obtained as a gift sample from Indochem Health Specialities Pvt. Ltd. Daman (India). Xanthan gum was a gift sample from SD. Fine Chemicals, Mumbai, and Capmul 908-P, Acconon MC-8-2 EP (polyoxyethylene (8) caprylic/capric glycerides) were gifted by Abitec Corporation, USA. Propylene glycol was purchased from SD. Fine Chemicals, Mumbai, India. Methanol (AR grade) was purchased from Loba Chemical Mumbai (India). Double distilled water was used for all experiments.

### 2.1. Solubility Studies

#### 2.1.1. Screening of Oils and Surfactants for Microemulsions

To find out suitable oil, surfactant, cosurfactant phase in microemulsions, the solubility of MTZ in various oils, surfactants, and cosurfactants were screened like Capmul MCM L, Capmul MCM L8, Capmul MCM C8, Capmul 908 P, Acconon MC8, Tween 80, Span 80, Tween 20, Caproyl 90, and Propylene glycol. An excess of MTZ was added individually to the oils, surfactants, and cosurfactant (5 g each) in screw capped tubes. Then the mixture was vortexed using a cyclomixer for 10 min in order to facilitate proper mixing of drug with the vehicles. Mixtures were then shaken for 48 h in a mechanical shaker (Remi, Mumbai, India) maintained at 25 ± 2°C. After 72 h, each sample was centrifuged at 5000 rpm for 10 min. The supernatant (0.5 mL) was diluted suitably, and the amount of MTZ present in the supernatant was analyzed by UV-spectrophotometer at 277 nm. The oil, surfactant, and cosurfactant phase that showed high solubility for MTZ were used in the preparation of microemulsions containing 1% MTZ.

#### 2.1.2. Construction of Phase Diagrams and Formulation of MTZ-Loaded Microemulsions

MTZ showed maximum solubility in Capmul 908 P as compared to other oils; hence it was selected for further studies. Acconon as a surfactant and propylene glycol as a cosurfactant showed better solubility for MTZ and good emulsifying properties with Capmul 908 P as oil phase. Pseudoternary phase diagrams were constructed using water titration method. Surfactants and cosurfactants (*S*_mix_) were mixed in different volume ratios (1 : 1, 2 : 1, 3 : 1, and 4 : 1). Oil and *S*_mix_ mixture were mixed thoroughly in different volume ratios (1 : 9, 1 : 8, 1 : 7, 1 : 6, 1 : 5, 1 : 4, 1 : 3, 1 : 2, and 1 : 1). Distilled water was added dropwise to the different mixtures of oil/*S*_mix_ till a cloudy dispersion was obtained. Pseudoternary plots were constructed using Chemix school trial version software 3.00, and microemulsions were prepared based on ternary phase diagram.

#### 2.1.3. Preparation of Emulgel

From preformulation studies xanthan gum was selected as the gel matrix to prepare the emulgel formulation. Xanthan gum was dispersed in purified water with constant stirring; the pH was adjusted from 6 to 6.5 using tri ethanol amine (TEA) [5]. Xanthan gum was slowly mixed with microemulsion in 1 : 1 ratio with constant stirring. After xanthan gum was entirely dissolved in the microemulsion, milky white emulgel was obtained (Table 1).

#### 2.1.4. Experimental Design and Statistical Analysis

2^3^ full factorial design was used to statistically optimize the formulation factors and evaluate main effects, interaction effects on the amount of MTZ permeated in 12 h, and spreadability [1, 2]. A 3-factor, 2-level factorial design was used to explore response surfaces and constructing second-order polynomial models with Design Expert software (Version 7.1, Stat-Ease Inc., Minneapolis, MN). The 2-level factorial design was specifically selected since it requires fewer runs than other experimental designs. A design matrix comprising 8 experimental runs was constructed. The nonlinear computer, generated quadratic model is given as

(1) Y=b0+b1X1+b2X2+b3X3+b12X1X2+b23X2X3+b123X1X2X3,

where *Y* is the measured response associated with each factor level combination; *b*_0_ is an intercept; *b*_1_ to *b*_123_ are regression coefficients computed from the observed experimental values of *Y*; and *X*_1_, *X*_2_, and *X*_3_ are the coded levels of independent variables. The terms *X*_1_, *X*_2_, and *X*_3_ (*i* = 1, 2  or  3) represent the interaction and quadratic terms, respectively. The dependent and independent variables were with their low and high levels, which were selected based on the results of pseudoternary phase diagrams. The proportion of oil (*X*_1_), *S*_mix_ (*X*_2_), and gelling agent (*X*_3_) used to prepare the 8 experimental trials and the respective observed responses are given in Table 2. All other formulation and processing variables were kept invariant throughout the study.

#### 2.1.5. Checkpoint Analysis and Optimization Model Validation

Statistical validation of the polynomial equations generated by Design Expert was established on the basis of ANOVA provision in the software. The models were evaluated in terms of statistically significant coefficients and *R* ^2^ values. The optimized formulations were selected on the basis of desirability based on acceptance criteria according to Design Expert software. Various 3D response surface graphs were provided by the Design Expert software. Three optimum checkpoint formulations were selected over the experimental domain to validate the experimental model and polynomial equations. The optimized checkpoint formulation factors were evaluated for various response properties. The resultant experimental values of the responses were quantitatively compared with the predicted values to calculate the percentage prediction error.

#### 2.1.6. Physical Appearance and pH Determination

The MTZ emulgels were inspected visually for their color, homogeneity, consistency, and pH. The pH values of 1% aqueous solutions of the emulgels were measured by a pH meter.

#### 2.1.7. Rheological Studies

The viscosity of the different emulgel formulations was determined at 25°C using a Brookfield viscometer (LV2, Brookfield Inc., USA) equipped with the *t*-bar spindle number 92, and the viscosities were recorded at different rotational speeds of 10, 20, 50, and 100 RPM.

#### 2.1.8. Spreading Coefficient

The spreading coefficient (spreadability) of the formulations was determined using an apparatus described by Jain et al. The apparatus consisted of two glass slides (7.5 × 2.5 cm), one of which was fixed onto the wooden board and the other was movable, tied to a thread which passed over a pulley, carrying a weight. Formulation (1 g) was placed between the two glass slides. Weight (100 g) was allowed to rest on the upper slide for 1 to 2 minutes to expel the entrapped air between the slides and to provide a uniform film of the formulation. The weight was removed, and the top slide was subjected to a pull obtained by attaching 30 g weight over the pulley. The time (sec) required for moving slide to travel a premarked distance (6.5 cm) was noted and expressed as spreadability. Spreadability is calculated by using the following formula:

(2) $$\begin{array}{c} S=\frac{M\cdot L}{T}, \end{array}$$

where *M* is weight tied to upper slide, *L* is length of glass slides, and *T* is time taken to separate the slides

#### 2.1.9. Drug Content Determination

MTZ content in emulgel was measured by dissolving known quantity of emulgel formulation in methanol by sonication. Absorbance was measured after suitable dilution at 277 nm using UV-Vis spectrophotometer.

#### 2.1.10. *In Vitro* Diffusion Studies

Franz diffusion cell was used for the drug diffusion studies. Emulgel (1 g) was evenly applied onto the surface of dialysis membrane. The dialysis membrane was clamped between the donor and the receptor chamber of diffusion cell. The receptor chamber was filled with freshly prepared phosphate buffer (pH 7.4). The receptor chamber was stirred by magnetic stirrer. The aliquots (1 mL) were collected at time intervals of 1 h up to 12 h. Samples were analyzed for drug content by UV-Vis spectrophotometer after appropriate dilutions. Cumulative corrections were made to obtain the total amount of drug release at each time interval. 

#### 2.1.11. *Ex Vivo *Diffusion Studies

*Ex vivo* diffusion study was carried out by using rat skin, and procedure was similar to that of *in vitro* diffusion study. Cumulative corrections were made to obtain the total amount of drug diffused at each time interval and *ex vivo* parameters were calculated. The average cumulative amount of drug permeated per unit surface area of the skin was plotted versus time [6]. The slope of the linear portion of the plot was calculated as flux *J*_ss_ (*μ*g/cm^2^/h), and the permeability coefficient was calculated using the following formula:

(3) $$\begin{array}{c} K_{p}=\frac{J_{\text{ss}}}{c_{v}}, \end{array}$$

where *K*_*p*_ is the permeability coefficient and *C*_*v*_ is the total amount of drug. 

The enhancement of drug penetration due to microemulsion formulation compared with marketed gel Metrogyl (J. B. Pharmaceuticals) was noted as enhancement factor (EF) [7] which was calculated using the following formula:

(4) $$\begin{array}{c} \text{EF}=\frac{K_{p}\left(\text{microemulsion}\;\text{based}\;\text{gel}\right)}{K_{p}\left(\text{MTZ}\;\text{gel}\right)}. \end{array}$$

#### 2.1.12. Skin Irritation Test

The skin irritation study was conducted in accordance with the approval of the Animal Ethical Committee, AISSMS College of Pharmacy (CPCSEA/IAEC/PT-03/12-2 K11), using white male rabbits (*n* = 3) as test animals. The hair of rabbits on dorsal side was shaved with electrical shaver and emulgel (about 4 gm) applied to each site (two sites per rabbit) by introduction under a double gauze layer on one square inch of the skin. After 24 h exposure, the formulation was removed. The test sites were wiped with tap water to remove any residual gel. The development of erythema/edema was monitored for 3 days by visual observation.

#### 2.1.13. Stability Studies

The stability studies were carried out as per the ICH guidelines. The emulgels were stored away from light in high-density polyethylene bottles at 40°C and 4°C for 3 months. After storage, the samples were tested for their physical appearance, pH, and drug content and drug release.

## 3. Results and Discussion

### 3.1. Selection of Excipients for Formulation of Microemulsions

The solubility of MTZ in various oils, surfactants and cosurfactant was analyzed in order to select components for microemulsions. MTZ is a BCS IV drug having extremely poor water solubility of (1 mg/mL). Due to poor solubility and permeability, microemulsions are attractive approaches to overcome bioavailability problems. Solubility of MTZ in various oils and *S*_mix_ was determined (Table 3). It was found that MTZ was found to have maximum solubility in Capmul 908 P, Acconon, and propylene glycol (35.14, 51.5, and 16.14 mg mL^−1^, resp.). Hence Capmul 908 P was selected as oil phase and Acconon, and propylene glycol was selected as surfactant and cosurfactant for further studies.

### 3.2. Construction of Pseudoternary Diagrams

**Figure 1:**
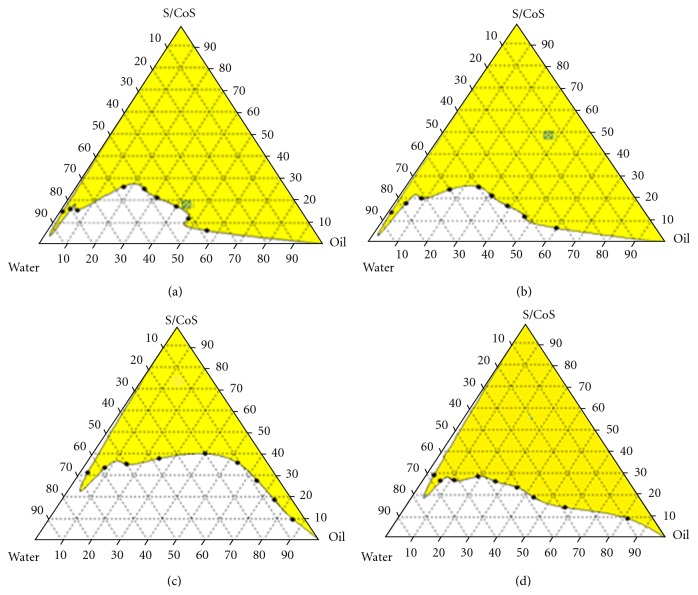
Pseudoternary phase diagrams of microemulsions composed of oil phase (Capmul 908 P), surfactant (Acconon), cosurfactant (propylene glycol), and water, where (a), (b), (c), and (d) were the different ratios of *S*_mix_ 1 : 1, 2 : 1, 3 : 1, and 4 : 1, respectively.

For the construction of pseudoternary phase diagrams, MEs, from the selected oil and surfactants, were prepared in different volume ratios (1 : 9, 1 : 8, 1 : 7, 1 : 6, 1 : 5, 1 : 4, 1 : 3, 1 : 2, and 1 : 1).  Figure 1 presents the pseudoternary phase diagrams with various weight ratios of Acconon/propylene glycol (1 : 1, 2 : 1, 3 : 1, and 4 : 1). From Figure 1, it was found that the ME area was maximum at *S*_mix_ ratio of 2 : 1. Hence this ratio was selected for preparation of drug-loaded MEs. At 1 : 1 ratio, the concentration of Acconon may not be sufficient to form a tightly packed barrier film. The ME region for 3 : 1 and 4 : 1 was significantly lesser than 1 : 1 and 2 : 1. Acconon is a C_8_ PEG-caprylic glyceride with HLB of 14 and molecular weight of 400 daltons. At higher concentration of Acconon, some of the molecules may be involved in formation of micelles. Micelles lie in the colloidal size range and hence may be contributing to the cloudiness of the dispersion. Thus we may presume that, for 3 : 1 and 4 : 1 ratios, reduced ME region is seen in the ternary plots. 

### 3.3. Physical Appearance and pH Determination

The MTZ emulgels were white viscous creamy preparation with a smooth homogeneous appearance. The pH values of all prepared formulation ranged from 6.0 to 6.9, which are considered acceptable to avoid the risk of irritation upon application to the skin because adult skin pH is 5.5. 

### 3.4. Rheological Studies

Rheological behavior of the emulgels indicated that the systems were shear thinning in nature showing decrease in viscosity at the increasing shear rates. The viscosity data has been summarized in Table 4. As the shear stress is increased, the normally disarranged molecules of the gelling material are caused to align their long axes in the direction of flow. Such orientation reduces the internal resistance of the material and hence decreases the viscosity [8]. An increase in the concentration of xanthan gum (1 to 3%) was expected to show increase in viscosity. However the microemulsions incorporated into the gel contained varying amounts of oil/*S*_mix_ which could be contributing to the viscosity of the formulations. Hence no particular trend was evident, though all formulations exhibited shear thinning properties. 

### 3.5. Spreading Coefficient

**Figure 2:**
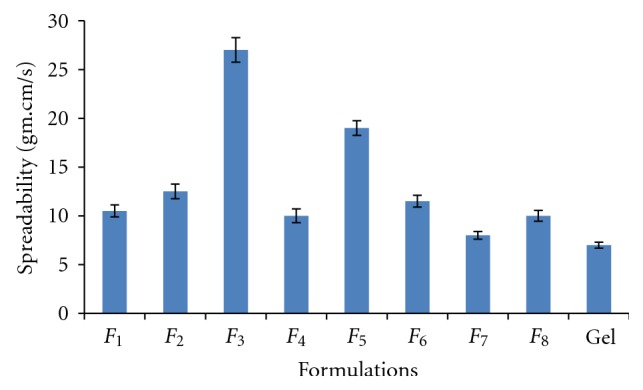
Spreadability of the *F*_1_ to *F*_8_  emulgel formulations (mean ± SD, *n* = 3).

One of the essential criteria for an emulgel is that it should possess good spreadability. Spreadability depends on the viscosity of the formulation and physical characteristics of the polymers used in the formulation. A more viscous formulation would have poor spreadability. Spreadability is a term expressed to denote the extent of area on which the gel readily spreads on application to the skin. The therapeutic efficacy of a formulation also depends upon its spreading value. The spreadability of different emulgel formulations is shown in Figure 2. It shows that the *F*_3_ formulation shows higher spreading coefficient as compared to other formulations.

### 3.6. *In Vitro *Diffusion Study

**Figure 3:**
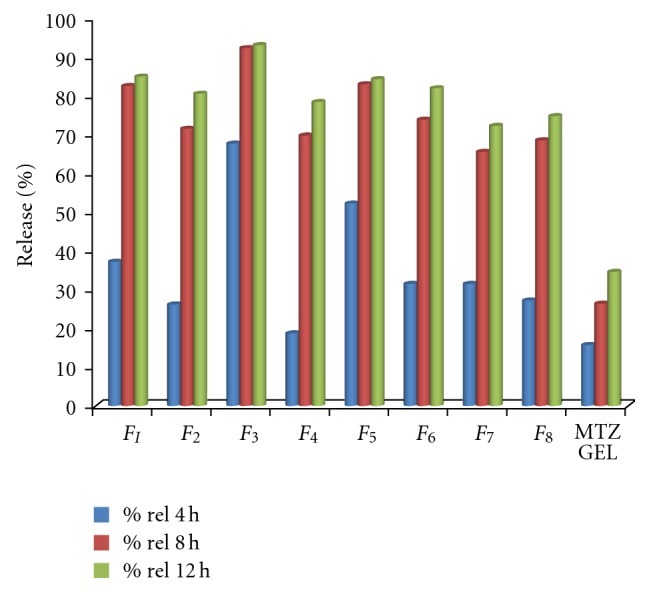
Cumulative percent of MTZ released from *F*_1_ to *F*_8_ emulgel formulations through dialysis membrane using Franz diffusion cell.

The *in vitro* diffusion profiles of MTZ from various emulgel formulations are represented in Figure 3. It was observed that all the formulation had become liquefied at the end of experiments, indicating water diffusion through the membrane. In general, it can be observed from the figures that all emulgels showed better release as compared to plain drug formulation. The higher drug release was observed with formulations *F*_3_ and *F*_5_. This finding may be due to presence of Capmul 908P in its low level and emulsifying agent in its high level. This led to an increase in the hydrophilicity of the emulgel, which in turn facilitated penetration of the release medium into the emulgel formulation. *F*_3_ and *F*_5_ formulations showed 93.16% and 83.06% cumulative drug permeation after 12 h. 

### 3.7. Formulation Optimization by Experimental Design

**Figure 4:**
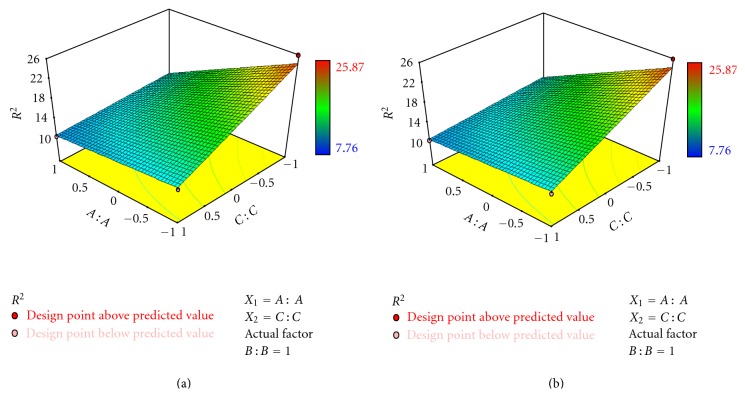
Response surface plot showing effect of (a) oil (*X*_1_) and (b) Gelling agent (*X*_3_) on responses percent drug release (*Y*_1_) and spreadability (*Y*_2_).

A three-factor, two-level full factorial experimental design was used to optimize the formulation variables as the response surface methodology requires 8 experiments. The independent variables and the responses for all 8 experimental runs are given in Table 2. The 3D response surface plots drawn using Design Expert software are shown in Figure 4. Based on the results of pseudoternary phase diagrams, appropriate ranges of the components were chosen. The oil phase concentration that could form microemulsion was found to be 10–70% and was selected as oil concentration to identify the optimum proportion of oil. Previous reports revealed that there was a really tight relationship between the hydration effect of the stratum corneum and the dermal permeation [9], and the thermodynamic activity of drug in microemulsions was a significant driving force for the release and penetration of drug into skin [9]. Based on pseudoternary phase diagrams, the surfactant mixture (surfactant, cosurfactant, and *S*_mix_ 2 : 1), that could form clear microemulsion with large area was selected as variable and was found to be 25–55%. Design Expert software was used to optimize the formulation and to develop the mathematical equations which are depicted in (5) and (6). The responses, percent drug diffusion (*Y*_1_) and spreadability (*Y*_2_) were found to be significantly higher (*Y*_1_, 93.16–83.06%; *Y*_2_, 25.87–19.54 gm·cm/sec) only when the oil and *S*_mix_ were used at 10% (v/v) and 55%  (v/v) concentration level, respectively. The ranges of other responses, *Y*_1_ and *Y*_2_ were 68.06–93.16% and 7.76–25.87 gm·cm/sec, respectively. The responses of these formulations ranged from a low drug diffusion of 68.60% (*F*_8_, high level of oil and *S*_mix_ and of high level of gelling agent) to a higher penetration of 93.16% (*F*_3_, low level of oil, high level of *S*_mix_, and low level of gelling agent). For estimation of quantitative effects of the different combination of factors and factor levels on percent drug diffusion and spreadability, the response surface models were calculated with Design Expert software by applying coded values of factor levels. The model described could be represented as

(5) Y1(percent  drug  diffusion) =81.36−2.80X1+1.5X2−0.25X3  −1.43X1X2−1.52X1X2X3,

(6) Y2(spreadability) =13.33−3.45X1+1.57X2−3.44X3  −2.49X1X2−1.23X1X2X3.

### 3.8. Fitting of Data to the Model

Formulation *F*_3_ showed a significantly higher amount of percent drug diffusion (*Y*_1_) and higher spreadability (*Y*_2_) among the formulations. The responses observed for 8 formulations prepared were simultaneously fit to design model, 2FI and 3FI models using Design Expert 7.1.5. It was observed that the best fit model was 3FI model, and the comparative values of *R* ^2^, standard deviation, and coefficient of variation (%) are given in Table 3 along with the regression equation generated for each response. A negative value represents an effect that favors the optimization, while a positive value indicates an inverse relationship between the factor and the response. It is evident that the independent variable *X*_3_ (concentration of gelling agent) was found to have a negative effect on the responses: percent dug diffusion (*Y*_1_) and spreadability (*Y*_2_). The independent variable *X*_2_ was found to have a positive effect on the percent dug diffusion (*Y*_1_) and spreadability (*Y*_2_). The three-dimensional response surface plots (Figure 4) were drawn to estimate the effects of the independent variables on response and to select the optimal formulation. 

### 3.9. Data Analysis

Formulations *F*_3_ and *F*_5_ had the higher percent drug diffusion and spreadability. The percent drug diffusion and spreadability obtained at various levels of the 3 independent variables (*X*_1_, *X*_2_, and *X*_3_) were subjected to multiple regression to yield a second-order polynomial equation. The value of the correlation coefficient (*R* ^2^) of (5) was found to be 0.9985, indicating good fit (Table 5). The “Pred *R*-Squared” of 0.9763 is in reasonable agreement with the “Adj *R*-Squared” of 0.9948. The percent drug diffusion values measured for the different formulations showed wide variation (i.e., values ranged from a minimum 68.06 to a maximum of 93.16%). The results clearly indicate that the percent drug diffusion is strongly affected by the variables selected for the study. The main effects of *X*_1_, *X*_2_, and *X*_3_ represent the average result of changing one variable at a time from its low level to its high level. The interaction terms show how the percent drug diffusion changes when two variables are simultaneously changed. The negative coefficients for all 3 independent variables indicate a favorable effect on the percent drug diffusion, while the positive coefficients for the interactions between 2 variables indicate an unfavorable effect on percent drug diffusion. 

The value of *R* ^2^ of (6) was found to be 0.9740, indicating good fit (Table 5). The “Pred R-Squared” of 0.8151 is in reasonable agreement with the “Adj *R*-Squared” of 0.9393. The spreadability values of *F*_3_ and *F*_5_ were found to be more among the formulations. The spreadability values were found to be increased from high to low levels of *X*_1_, low to high levels of variable *X*_2_, and low levels of *X*_3_. The spreadability values measured for the different formulations showed wide variation (i.e., values ranged from a minimum of 7.76 in *F*_4_ to a maximum of 25.87 in *F*_3_). The interaction terms show how the spreadability changes when 2 variables are simultaneously changed. The negative coefficients *X*_1_ and *X*_3_ for the interactions between 2 variables indicate a favorable effect on spreadability. 

### 3.10. Validation of Response Surface Methodology

**Figure 5:**
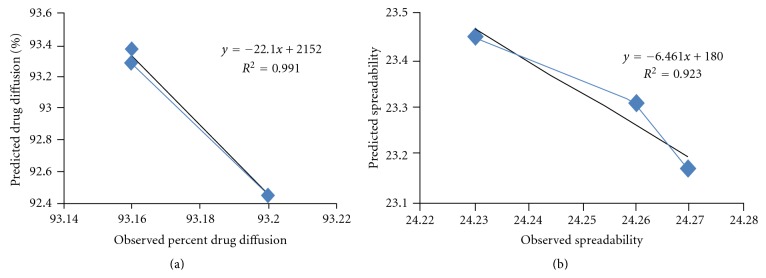
Linear correlation plots ((a) and (b)) between actual and predicted values.

Three checkpoint formulations were obtained from the RSM, the composition, and predicted responses which are listed in Table 6. To confirm the validity of the calculated optimal parameters and predicted responses, the optimum formulations were prepared according to the above values of the factors and subjected to *ex vivo* permeation studies. From the results presented in Table 5, the predicted error was below 5%, indicating that the observed responses were very close to the predicted values. Percentage prediction error is helpful in establishing the validity of generated equations and to describe the domain of applicability of RSM model. Linear correlation plots between the actual and the predicted response variables were shown in Figure 5. The linear correlation plots drawn between the predicted and experimental values demonstrated high values of *R* ^2^ (percent drug diffusion, 0.9919; spreadability, 0.9231) indicating goodness of fit. 

### 3.11. *Ex Vivo* Diffusion Study

**Figure 6:**
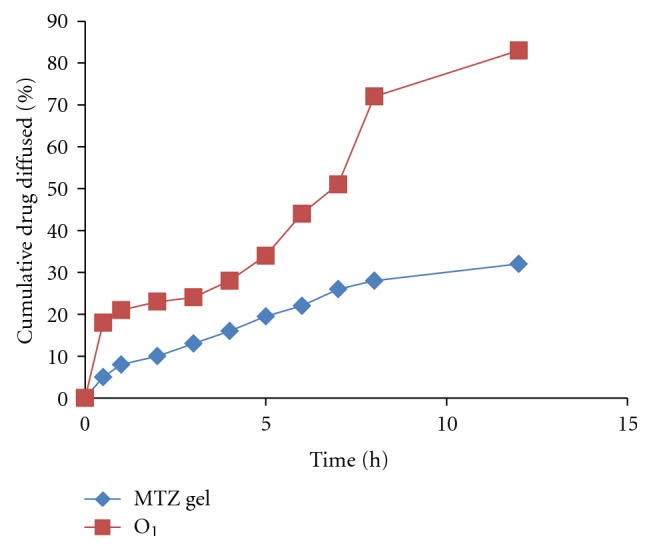
Cumulative amount of Metronidazole diffused (in %) from the optimized emulgel and MTZ gel through rat skin using Franz diffusion cell.

The *ex vivo* release study of optimized emulgel (10% oil, 55% *S*_mix_, and 1% gelling agent) compared with the 1% MTZ gel formulation. The optimized emulgel and MTZ gel showed the 83.14% and 34.63% release at the end of 12 h, respectively. The emulgel exhibited higher flux and permeation coefficient as compared to the MTZ gel formulation (Figure 6). The results showed that the MTZ emulgel has the steady state flux (*J*_*ss*_) 351.78 (*μ*g/cm^2^/h) and apparent permeation coefficient (*K*_*p*_) 35.17 (cm/h) × 10^−3^ (Table 7). The permeability enhancement factor for emulgel when compared with marketed formulation Metrogyl was found to be 3.65. 

### 3.12. Skin Irritation Test

The skin irritation studies were carried out to evaluate the tolerability of the emulgel components after application. It was observed that emulgels were very well tolerated by the rabbits, and no signs of erythema and/or edema were seen even after 3 days.

### 3.13. Stability Studies

Short-term accelerated stability of emulgel was found after 3 months at 40°C/75% RH and 4°C. Emulgels were found to be white viscous creamy preparation with the smooth homogenous appearance which is similar to the day on which it was formulated. pH and the drug release of formulation were found to be 6.3 ± 0.4 and 82.7 ± 1.02%, respectively. Thus the formulations were found to be stable under accelerated conditions. There was no evidence of syneresis in the emulgels which is a common drawback of gels.

## 4. Conclusion

The present study conclusively demonstrates that the use of a 2^3^ full factorial design is valid for predicting the percent drug diffusion and spreadability in optimization of emulgel formulations. The derived polynomial equations and contour plots aid in predicting the values of selected independent variables for preparation of optimum emulgel with desired properties. The developed emulgels were efficacious for the delivery of lipophilic and poorly soluble drugs such as Metronidazole. The results demonstrated that the formulations were stable and showed improved permeation of the drug from the emulgel compared to conventional gel.

## Figures and Tables

**Table 1:** Formulation of various emulgels.

Ingredients (%w/w)	*F*_1_	*F*_2_	*F*_3_	*F*_4_	*F*_5_	*F*_6_	*F*_7_	*F*_8_
MTZ	1.00	1.00	1.00	1.00	1.00	1.00	1.00	1.00
Capmul 908 P	70	10	10	10	10	70	70	70
Acconon MC 8-2 EP	16.67	36.67	16.67	36.67	16.67	16.67	36.67	36.67
Propylene glycol	8.33	18.33	8.33	18.33	8.33	8.33	18.33	18.33
Methyl paraben	0.2	0.2	0.2	0.2	0.2	0.2	0.2	0.2
Propyl paraben	0.02	0.02	0.02	0.02	0.02	0.02	0.02	0.02
Triethanolamine	q.s	q.s	q.s	q.s	q.s	q.s	q.s	q.s
Xanthan gum	3	1	1	3	3	1	3	1
Water	q.s	q.s	q.s	q.s	q.s	q.s	q.s	q.s

**Table 2:** Variables and observed responses in 2^3^ factorial design for emulgel formulations (mean ± SD, *n* = 3).

Formulations	Independent variables			Dependent variables	
	*X*_1_	*X*_2_	*X*_3_	*Y*_1_	*Y*_2_
*F*_1_	1	−1	−1	81.03	9.74
*F*_2_	− 1	1	1	80.64	11.66
*F*_3_	−1	1	−1	93.16	25.87
*F*_4_	1	1	1	74.85	7.76
*F*_5_	−1	−1	−1	84.37	19.54
*F*_6_	1	1	−1	82.04	11.96
*F*_7_	−1	−1	1	78.49	10
*F*_8_	1	−1	1	72.33	10.12

Independent variables	Levels used, actual (coded)				
	Low (−1)			High (1)	

*X*_1_= conc. of oil	10%			70%	
*X*_2_= conc. of *S*_mix_	25%			55%	
*X*_3_= conc. gelling agent	1%			3%	

**Table 3:** Solubility data of MTZ in different oils, surfactants, and cosurfactants (mean ± SD, *n* = 3).

Sr. no.	Name of oil	Solubility (mg/mL) ± SD
1	Capmul 908 P	35.14 ± 1.54
2	Capmul MCM L	10.2 ± 0.98
3	Labrafac	13.45 ± 1.23
4	Capmul MCM C-8	1.86 ± 0.44
5	Capmul MCM L-8	2.05 ± 0.87
6	Capmul MCM	1.77 ± 0.53
7	Captex 100	0.85 ± 0.37
8	Span 80	1.98 ± 0.68
9	Tween 80	8.45 ± 0.89
10	Tween 20	27.62 ± 1.89
11	Labrasol	17.10 ± 0.57
12	Acconon MC8-2	51.5 ± 2.24
13	Transcutol	8.12 ± 0.54
14	Capryol-90	8.96 ± 0.46
15	PEG 400	10.88 ± 0.69
16	Propylene glycol	16.14 ± 1.07

**Table 4:** Viscosities of the emulgel formulations at different rotational speeds (mean ± SD, *n* = 3).

RPM	Viscosity (mPas)							
	*F*_1_	*F*_2_	*F*_3_	*F*_4_	*F*_5_	*F*_6_	*F*_7_	*F*_8_
10	4232 ± 0.53	4856 ± 0.45	4568 ± 0.32	4314 ± 0.76	3622 ± 0.47	4462 ± 0.56	4636 ± 0.65	5294 ± 0.76
20	2562 ± 0.21	2267 ± 0.87	2165 ± 0.11	2087 ± 0.65	2265 ± 0.53	2901 ± 0.67	2134 ± 0.87	3421 ± 0.65
50	1567 ± 0.32	1237 ± 0.65	1187 ± 0.38	1347 ± 0.45	1463 ± 0.58	1674 ± 0.34	1098 ± 0.54	1678 ± 0.87
100	970 ± 0.75	1198 ± 0.54	1087 ± 0.43	1298 ± 0.34	1209 ± 0.69	1380 ± 0.32	965 ± 0.98	1567 ± 0.64

**Table 5:** Summary of results of regression analysis for responses *Y*_1_ and *Y*_2_.

3FI model	*R* ^2^	Adjusted *R* ^2^	Predicted *R* ^2^	SD	% CV
Response (*Y*_1_)	0.9985	0.9948	0.9763	0.47	0.57
Response (*Y*_2_)	0.9740	0.9393	0.8151	1.09	1.47

**Table 6:** Composition of checkpoint formulations, the predicted, and experimental values of response variables and percentage prediction error.

Formulation code	Composition (%w/w)	Response	Predicted value	Experimental value	Percentage error
O_1 _	10∗	Release (%)	93.20	92.45	−0.80
	55^#^	Spreadability	24.27	23.97	−0.98
	1^$^				

O_2 _	10∗	Release (%)	93.16	93.48	+0.12
	30.85^#^	Spreadability	24.26	23.91	−1.04
	1^$^				

O_3_	0.9∗	Release (%)	93.16	93.08	−0.23
	47.73^#^	Spreadability	24.23	23.90	−1.36
	1^$^				

∗Concentration of oil, ^#^concentration of *S*_mix_, and ^$^concentration of gelling agent.

**Table 7:** Comparison of diffusion parameters of optimized formulation with MTZ gel (mean ± SD, *n* = 3).

Formulation	*J*_SS_ (*μ*g/cm^2^/h)	*K*_*P*_ (cm/h) ×10^−3^	Cum. amt. permeated at 12 h (*μ*g/cm^2^)
Optimized emulgel	351.78 ± 2.23	35.17 ± 2.45	2589.17 ± 4.21
MTZ GEL	96.27 ± 2.54	9.62 ± 2.64	1102.86 ± 4.65
